# Better quality of life with neuropsychological improvement on HAART

**DOI:** 10.1186/1477-7525-4-11

**Published:** 2006-02-24

**Authors:** Thomas D Parsons, Alyssa J Braaten, Colin D Hall, Kevin R Robertson

**Affiliations:** 1 AIDS Neurological Center, University of North Carolina at Chapel Hill, 3114 Bioinformatics Building, Chapel Hill, NC 27599-7025, USA

## Abstract

**Background:**

Successful highly active antiretroviral therapy (HAART) regimens have resulted in substantial improvements in the systemic health of HIV infected persons and increased survival times. Despite increased systemic health, the prevalence of minor HIV-associated cognitive impairment appears to be rising with increased longevity, and it remains to be seen what functional outcomes will result from these improvements. Cognitive impairment can dramatically impact functional ability and day-to-day productivity. We assessed the relationship of quality of life (QOL) and neuropsychological functioning with successful HAART treatment.

**Methods:**

In a prospective longitudinal study, subjects were evaluated before instituting HAART (naïve) or before changing HAART regimens because current therapy failed to maintain suppression of plasma viral load (treatment failure). Subjects underwent detailed neuropsychological and neurological examinations, as well as psychological evaluation sensitive to possible confounds. Re-evaluation was performed six months after institution of the new HAART regimen and/or if plasma viral load indicated treatment failure. At each evaluation, subjects underwent ultrasensitive HIV RNA quantitative evaluation in both plasma and cerebrospinal fluid.

**Results:**

HAART successes performed better than failures on measures exploring speed of mental processing (p < .02). HAART failure was significantly associated with increased self-reports of physical health complaints (p < .01) and substance abuse (p < .01). An interesting trend emerged, in which HAART failures endorsed greater levels of psychological and cognitive complaints (p = .06). Analysis between neuropsychological measures and QOL scores revealed significant correlation between QOL Total and processing speed (p < .05), as well as flexibility (p < .05).

**Conclusion:**

Our study investigated the relationship between HIV-associated neurocognitive impairment and quality of life. HAART failures experienced slower psychomotor processing, and had increased self-reports of physical health complaints and substance abuse. Contrariwise, HAART successes experienced improved mental processing, demonstrating the impact of successful treatment on functioning. With increasing life expectancy for those who are HIV seropositive, it is important to measure cognitive functioning in relation to the actual QOL these individuals report. The study results have implications for the optimal management of HIV-infected persons. Specific support or intervention may be beneficial for those who have failed HAART in order to decrease substance abuse and increase overall physical health.

## Background

Cognitive impairments are known to be associated with human immunodeficiency virus (HIV) infection. In HIV-1-associated minor cognitive/motor disorders patient profiles are characterized by impaired motor speed and working memory [1]. Contrariwise, attention, visuo-constructive abilities, and memory are relatively unimpaired [2-4]. In HIV-1-associated dementia, behavioral changes, attention and executive dysfunction, psychomotor slowing, and memory impairment mark patient profiles [5]. Although highly active antiretroviral therapy (HAART) has reduced the incidence of HIV dementia, HIV-associated cognitive impairment continues to be a major clinical problem among individuals with advanced infection [6]. Given the large number of potential pathways by which HIV-1 enters brain tissue, persists, and is activated over time, there is a significant possibility that HIV-1-associated cognitive-motor disorders may yet increase as a consequence of increasing resistance to HAART regimens [7]. Moreover, individuals with HIV-1 infection may experience various neurobehavioral changes. For example, the debilitating nature of the HIV disease course has been found to be associated with increased depression and anxiety, as well as poor quality of life [8].

Successful HAART regimens have resulted in substantial improvements in the systemic health of patients with HIV infection and increased survival times. However, with the increased longevity of HIV patients, the prevalence of minor HIV-associated cognitive impairment appears to be rising among HAART successes. HAART has been shown to successfully restore immune function and reduce the effects of opportunistic infection. This restoration of immune function results in a marked improvement of HIV-associated diseases and in the reduction of AIDS-related mortality [9]. However, although HAART improves physical health, the treatment's effects on neurocognitive and affective symptoms are unclear. Some studies have shown significant improvement in cognitive functioning [10,11], while others have shown that some patients continue to exhibit neurocognitive impairment even after extended periods of HAART [12,13]. In one study HIV-associated neurocognitive impairment was found to be present in nearly one-third of patients on HAART [14].

In addition to neurocognitive deficits, patients afflicted with HIV often exhibit affective disorders such as depression and anxiety. In fact, lower quality of life scores have been found to be associated with a diagnosis of HIV and with disease-related symptoms [8,15-17]. Neurocognitive and affective symptoms appear to be directly related, as patients exhibiting deficits on neuropsychological testing also report increased levels of depression and/or anxiety on self-report measures. This relationship is likely explicated by the fact that cognitively impaired patients are less likely to employ effective strategies to manage stressors and in turn to alleviate symptoms of depression and anxiety [18]. HAART seems to lessen the affective symptoms associated with HIV infection [19,20], likely due to the reduction of physical symptoms associated with the disorder.

Quality of life in HIV infection has been shown to be directly associated with disease stage, disease symptoms, and cognitive function [14]. Specifically, impairment in cognitive abilities including fine motor functions, memory, mental flexibility, concentration, speed of mental processing, visuospatial abilities, and constructional abilities correlate with reduced quality of life [18]. This finding reinforces the fact that patient's perceptions of their quality of life is related to their ability to function in society and their ability to succeed in activities of daily living [18]. Further, quality of life has been found to improve with antiretroviral therapy [21,22]. Significant improvements in the quality of life of symptomatic HIV subjects have been noted on quality of life measures following antiretroviral therapy [23]. Thus, a combination of cognitive functioning, affective disturbance, and physical symptoms need to be considered when assessing patient's quality of life and the overall functional outcome of HAART treatment.

While the effects of HAART have resulted in substantial improvements in the systemic health of patients with HIV infection, it remains to be seen what functional outcomes will result from these improvements. Impairment in cognition can have dramatic impacts on the ability to function on a day-to-day basis, and to be productive. Initial studies have demonstrated that neurocognitive improvement occurs in most patients on HAART therapy. In this study, we assess the relationship of quality of life and neuropsychological functioning with successful HAART treatment.

## Methods

The University of North Carolina Institutional Review Board approved the study, and all participants gave informed consent for participation. Participants were enrolled in the study under the following conditions: 1) if they were about to start HAART; or 2) if, in the opinion of their infectious disease clinician, their current HAART had failed and they required a different HAART regimen. There was no requirement for (or stratification by) presence of neurologic disease at study entry. Prior to starting or changing an antiretroviral regimen, a baseline evaluation was conducted. Participants were reevaluated after 6 months of stable HAART or when, in the opinion of the treating infectious disease physician, the new regimen failed. A neurologist conducted a quantified previously validated examination particularly sensitive to the changes found in HIV disease at each evaluation. At each evaluation, a neuropsychologist conducted detailed psychological and neuropsychological evaluations that have also been validated as sensitive to the neurocognitive changes found in HIV disease. At each evaluation, subjects also underwent ultrasensitive HIV RNA quantitative evaluation of both plasma and cerebrospinal fluid.

### HIV RNA Load

Viral load assessment was performed through plasma and cerebrospinal fluid measurements. Within eight hours of the neurologic evaluation, specimens for viral load and immune functioning were obtained. Blood and cerebrospinal fluid samples were obtained within 3 hours of each other. Cerebrospinal fluid samples were centrifuged to remove cells. The Roche Ultrasensitive assay was used to measure Quantitative HIV-1 RNA load. Prior to viral load analysis, neurologic and neuropsychological evaluations were completed in a blinded fashion.

### Neurologic examination

The Price and Sidtis [24] AIDS Clinical Trials Group full neurologic evaluation was used. The neurologic evaluation includes a global assessment of HAD (AIDS dementia complex stage), which varies from equivocal (0.5) to severe (3.0) dementia. To increase the sensitivity of the instrument and to provide domains of functioning, a quantitative scoring procedure for the neurologic evaluation was implemented, which provides a weighted scoring approach to the items of the neurologic examination and yields an overall neurologic total score as well as scores for cognitive, frontal, pyramidal, extrapyramidal, cranial nerve, cerebellar, spinal, autonomic, sensory, and peripheral domains.

### Neuropsychological evaluation

The neuropsychological evaluation included assessment of the following domains: attention/concentration (2 and 7 test, PASAT), speed of processing (computerized simple and choice reaction time tasks, digit symbol, trailmaking A, stroop word), executive functioning (trailmaking B, stroop colorword, COWA), visuospatial (Rey complex figure copy), verbal memory (RAVLT), figural memory (Rey complex figure immediate, delay), gross motor (timed gait), fine motor (grooved pegboard, finger tapping), and language (WAIS-R Vocabulary).

### Quality of Life

The Neurological Quality of Life questionnaire (NeuroQOL) is a self report instrument which assesses eleven domains: Security, Food, Housing, Financial, Productivity, Social support, Relationships, Psychological health, Physical health, Substance abuse, and Cognitive/Neurological problems. The NeuroQOL questionnaire contains 114 items answered in Likert format. The items are summed for a total score, with higher scores reflecting better quality of life [25,26].

### Memorial Sloan Kettering dementia staging

Subsequent to each visit, the Memorial Sloan Kettering dementia scale was determined for each participant at a consensus conference, utilizing the neurologic and neuropsychological evaluations, the psychiatric interview, and the quality of life scale. The Memorial Sloan Kettering dementia scale is as follows: normal, 0; equivocal (deficits that do not reach the stage of clear dementia), 0.5; and dementia, 1-3 (depending on severity) [24].

HAART treatment success and failure

We defined success and failure on HAART by both virological and immunological changes. We defined virological success as a decrease in plasma HIV RNA by 1.5 log copies/ml and to less than 2.5 log copies/ml. Virological failures were defined as a decrease of less then 1.5 log copies/ml or a failure to reduce HIV RNA below 2.5 log copies/ml. We defined immunological success as an increase in CD4+ cell count. Immunological failure was defined as stable or decreased absolute CD4+ cell count.

### Data analysis

First, HAART treatment and results on the neuropsychological battery were compared between groups (HAART success group and HAART failure group) by using t tests and an alpha level of 0.05. Next, HAART treatment and results on the NeuroQol were compared between groups (HAART success group and HAART failure group) by using t tests and an alpha level of 0.05. Pearson product-moment correlation was used to analyze the relationship between performance on the neurocognitive tasks and NeuroQol domains.

## Results

Eighty-six subjects were evaluated at baseline before treatment with HAART or after failing one treatment regimen and before instituting a different HAART regimen. Fifty nine of those then underwent six-month follow-up on stable HAART. As an ongoing study with continuous enrollment, seven of these participants did not have opportunity to attend their 6-month followup, as the data base was cut prior to their 6-month follow up. The dropout characteristics of the remaining participants include: five participants refused lumbar puncture, five had illicit drug problems, 2 never started HAART, 2 went to prison, 2 had other health related problems, and 2 moved out of the area. Of those with follow-up (N = 59), the median age was 42.52 years (SD = 7.58) and a median educational level of 12 years (SD = 2.15 years). The median CD4+ cell count was 290 (SD = 242.89), and the median plasma HIV RNA was 3.04 (SD = 1.84). Demographic, virologic, and immunologic variables are listed on Table 1.

**Table 1:** Demographic, virologic, and immunologic variables

**Variable**	**Baseline**	**During HAART**
Age (y)	41.00 ± 7.77	42.52 ± 7.58
Education (y)	12.00 ± 2.19	12.00 ± 2.15
Plasma HIV level (log copies/mL)	4.87 ± 1.41	3.04 ± 1.84
CSF HIV level (log copies/mL)	3.04 ± 1.47	1.41 ± 1.23
CD4+ cell count (/mm3)	203 ± 183.47	290 ± 242.89

Note: For all analyses N = 59. Data are mean ± SD; CSF = cerebrospinal fluid

Forty (68%) subjects were male and 19 were female (32%). Forty-four (75%) were black, 13 (22%) white, 1 was Asian and 1 was Native American. Forty (68%) had AIDS by CDC criteria, 7 (12%) were symptomatic and 12 (20%) were asymptomatic.

A quantitative scoring procedure was utilized for the neurological exam, and summary z-scores were calculated for the neuropsychological battery.

Participants with successful HAART treatment performed better than failure patients on measures exploring speed of mental processing (t = -2.46; p < .02). For complete results comparing Success and Failure groups on neuropsychological measures, see Table 2. The presence of HAART failure was significantly associated with increased self-reports of physical health complaints (t = 2.64; p < .04). Further, there was an interesting trend, in which the HAART failure group endorsed greater levels of psychological (t = 1.85; p = .07) and cognitive complaints (t = 1.88; p = .06). For complete results comparing Success and Failure groups on quality of life measures, see Table 3. Analysis between neuropsychological measures and quality of life scores revealed significant correlation between NeuroQOL Total and processing speed (r = -.27; p < .01), as well as flexibility (r = -.24; p < .03). For complete correlational results see Table 4.

**Table 2:** Comparison of success and failure groups on neuropsychological tests

	**Failure Mean**	**Success Mean**	**t-value**	**p**
Attention	-0.14	-0.22	0.33	0.74
Speed	-0.78	0.09	-2.46	**0.02**
Flexibility	-0.59	-0.61	0.07	0.95
Visual	-0.52	-0.40	-0.33	0.74
Verbal	-0.52	-0.63	0.45	0.65
Figural	-0.23	-0.39	0.78	0.44
Fine Motor	-0.54	-0.08	-1.14	0.26
Gross Motor	-0.24	0.12	-0.46	0.65
Language	-0.21	0.08	-1.22	0.23
Neuropsychology Total Z-score	-0.52	-0.21	-1.48	0.14

Note: For all analyses N = 59.

**Table 3:** Comparison of success and failure groups on NeuroQOL

	**Failure Mean**	**Success Mean**	**t-value**	**p**
Food	4.63	2.78	1.20	0.23
Housing	9.44	4.56	1.66	0.10
Financial	3.91	0.84	1.28	0.20
Product	5.91	4.06	0.62	0.54
Social	6.17	3.47	0.98	0.33
Relation	7.80	6.88	0.27	0.79
*Psych*	*26.78*	*9.38*	*1.85*	*0.07*
Physical	18.02	2.50	2.64	**0.01**
Substance	7.67	0.06	2.80	**0.01**
*Cognitive*	*12.19*	*3.59*	*1.88*	*0.06*
NeuroQOL Total	106.98	41.53	1.82	0.07

Note: For all analyses N = 59. NeuroQOL Total is the total score for the Quality of Life measure.

Mean = 82.63. Standard deviation = 163.47

**Table 4:** Correlations between NeuroQOL total and neuropsychological measures

	**Mean**	**Std.Dv.**	**r(X, Y)**	**P**
Attention	-0.17	1.11	0.01	Ns
Speed	-0.45	1.64	-0.27	0.01
Flexibility	-0.60	1.64	-0.24	0.03
Visual	-0.48	1.69	0.03	Ns
Verbal	-0.56	1.08	0.23	Ns
Figural	-0.29	0.89	0.13	Ns
Fine Motor	-0.37	1.79	0.08	Ns
Gross Motor	-0.11	3.48	0.34	Ns
Language	-0.10	1.09	0.74	Ns

Note: For all analyses N = 59. NeuroQOL Total is the total score for the Quality of Life measure.

Mean = 82.63. Standard deviation = 163.47

## Discussion

Our study investigated the relationship between HIV-associated neurocognitive impairment and quality of life. Our findings revealed that subjects who failed HAART experienced slower psychomotor processing, and had increased self-reports of physical health complaints and substance abuse. On the other hand, those with successful HAART experienced improved mental processing, demonstrating the impact of successful treatment on functioning.

Restoration of immune function results in a marked improvement of HIV-associated diseases and in the reduction of AIDS-related mortality [9]. With increasing life expectancy for those who are HIV seropositive, it is important to measure cognitive functioning in relation to the actual quality of life these individuals report. Our results revealed that participants with successful HAART treatment performed better than failure patients on measures exploring speed of mental processing, which reflects the current literature [27]. We also found significant correlations between total quality of life and processing speed, as well as flexibility.

The presence of HAART failure was significantly associated with increased self-reports of physical health complaints. These findings reflect current literature, in which lower quality of life scores have been found to be associated with a diagnosis of HIV and with disease-related symptoms [8,15-17]. Further, there was an interesting trend, in which the HAART failure group endorsed greater levels of psychological and cognitive complaints. These findings are consistent with findings that neurocognitive and affective symptoms appear to be directly related, as patients exhibiting deficits on neuropsychological testing also report decreased quality of life. This relationship is likely explicated by the fact that cognitively impaired patients are less likely to employ effective strategies to manage stressors and in turn to alleviate symptoms of depression and anxiety [18].

Quality of life in HIV infection has been shown to be directly associated with disease stage, disease symptoms, and cognitive function [14,28]. Specifically, impairment in cognitive abilities including fine motor functions, memory, mental flexibility, concentration, speed of mental processing, visuospatial abilities, and constructional abilities correlate with reduced health-related quality of life [18]. Our findings reinforce the fact that patient's perceptions of their quality of life is related to their ability to function in society and their ability to succeed in activities of daily living [18]. Thus, a combination of cognitive functioning, affective disturbance, and physical symptoms need to be considered when assessing patient's quality of life and the overall functional outcome of HAART treatment.

The study results have implications for the optimal management of HIV-infected patients' neurocognitive impairment. Our findings support continued investigation of the presence of neurocognitive impairment in the HAART era. The association we found with poor quality of life scores further emphasizes this need. Given these findings, specific support or intervention may be beneficial for those who have failed HAART in order to decrease substance abuse and increase overall physical health. As a subjective evaluation of persons with HIV related illness, quality of life provides information relevant to patient care, and health promoting activities. Specific support or intervention may be beneficial for those who have failed HAART in order to increase a person's ability to achieve and maintain a level of overall functioning necessary to decrease substance abuse and increase overall physical health. The identifying of factors that diminish quality of life in HIV cases is an important step towards improving quality of life in this population. Further, it allows for screening of these factors, and in situations where these factors are present, it aides the clinician in planning and implementing interventions.

Future studies should look at the relations among quality of life, adherence, substance abuse, neurocognitive scores, and virological and immunological outcome. Adherence is a critical component for therapeutic success in HIV infection and has been shown to be a major determinant of biological outcome measures in HIV. Substance abuse has been associated with decreased adherence in several studies [29,30]. Divergent findings have resulted from studies investigating adherence and quality of life [31-33]. The relationship between quality of life, adherence, and neurocognitive sequelae has not been well studied. Hence, future studies should look at the interrelations among quality of life, adherence, substance abuse, neurocognitive scores, and virological and immunological outcome.

## Conclusion

In summary, our assessment of quality of life and neuropsychological functioning with HAART treatment revealed that HAART success was significantly related to processing speed. HAART failure was significantly associated with increased physical health complaints, substance abuse, and a trend toward increased psychological and cognitive complaints. Significant correlations were found between quality of life and processing speed, as well as flexibility. Future studies should include risk and/or severity factors and look at the relationship between quality of life, adherence, and neurocognitive sequelae.

## Authors' contributions

TDP, KRR and CDH planned the study and collected the data. KRR identified the patient cohort and collected the data. TDP performed the statistical analysis and drafted the manuscript together with KRR, CDH, and AJB (TDP, KRR, CDH, and AJB contributed equally to this study). All authors read and approved the final manuscript.
